# Associations between residential fossil fuel combustion and indoor concentrations of nitrogen dioxide, carbon monoxide, and aldehydes in Canadian homes

**DOI:** 10.1038/s41370-025-00762-6

**Published:** 2025-03-14

**Authors:** Liu Sun, Marie-Ève Héroux, Xiaohong Xu, Amanda J. Wheeler

**Affiliations:** 1 Water and Air Quality Bureau, Health Canada, Ottawa, ON Canada; 2 Department of Civil and Environmental Engineering, University of Windsor, Windsor, ON Canada; 3 Menzies Institute for Medical Research, University of Tasmania, Hobart, TAS Australia; 4 Commonwealth Scientific and Industrial Research Organization, Aspendale, VIC Australia

**Keywords:** Indoor air quality, Fossil fuel combustion, Gas stove cooking, Nitrogen dioxide, Carbon monoxide, Aldehydes

## Abstract

**Background:**

There is increasing attention on the effects of residential fossil fuel combustion, particularly the use of natural gas or oil, on indoor air quality. Given the prevalent use of natural gas in Canadian homes, understanding its influence on indoor air quality is important.

**Objective:**

This study investigated associations between indoor levels of nitrogen dioxide (NO_2_), carbon monoxide (CO), formaldehyde, and acetaldehyde with potential emission sources and other influencing factors in 344 homes in four Canadian cities.

**Methods:**

Using mixed models and general linear models, we evaluated the associations between potential sources and pollutant concentrations, conducting both city-specific and pooled analyses for winter and summer seasons.

**Results:**

Our findings indicated that gas stoves, present in 24% of the homes, were significantly associated with increased indoor NO_2_ concentrations, resulting in a 191% increase in winter and a 114% increase in summer. Additionally, the presence of gas stoves was strongly associated with a 43% increase in peak hourly CO levels in winter. The presence of gas clothes dryers was significantly associated with increased indoor NO_2_ levels (47% in summer and 54% in winter). Oil heating was significantly associated with a 58% increase in winter indoor NO_2_ levels. Gas heating was associated with a 62% increase in winter NO_2_ levels in older homes (built before 1949), with marginal significance. Aldehyde levels were primarily associated with off-gassing from building materials and household activities. Other factors associated with indoor pollutant levels included housing characteristics, occupant behaviors, indoor environmental conditions, and outdoor sources.

**Impact:**

- This study enhances understanding of the association between fossil fuel combustion and indoor air quality in predominantly detached homes. It highlights differences in pollutant levels between homes with gas and electric cooking, which can inform advice on cooking practices to reduce emissions in homes.

## Introduction

There is increasing attention on the effects of residential fossil fuel combustion on indoor air quality (IAQ), particularly the use of natural gas or heating oil. In Canadian residences, natural gas, hereafter referred to as “gas”, is the predominant energy source, accounting for 45% of total household energy consumption [1]. It is used for home and water heating, as well as gas-powered appliances such as stoves, ovens, fireplaces, and clothes dryers. Heating oil, a type of fuel oil used for home heating, is prevalent in regions of Prince Edward Island and Nova Scotia. The combustion of fossil fuels releases a range of air pollutants, including nitrogen dioxide (NO₂), carbon monoxide (CO), and aldehydes, particularly formaldehyde and acetaldehyde [2–7].

While pollutants from gas or oil heating systems are typically vented to the outdoors, exposures from gas stoves and ovens depend on the effectiveness of range hoods, which varies and depends on user behavior [5, 8–11]. Additionally, the inadequate use of range hoods in Canadian homes, mainly attributed to noise and low perceived effectiveness, increases the risk of exposure to cooking pollutants [12]. Given that Canadians spend about 90% of their time indoors [13], understanding the impact of residential fossil fuel combustion on IAQ is important.

Although many studies have examined emissions from combustion appliances in both laboratory and residential settings [5, 7, 14–19], research on the impact of combustion sources, particularly unvented gas appliances such as stoves and ovens, on indoor pollutant levels of NO_2_, CO, and aldehydes in Canadian homes is limited. The unique climatic and structural conditions in Canada can significantly influence indoor air quality dynamics, highlighting the need for research specific to these conditions. One study by Gilbert et al. [20] reported notably higher concentrations of NO_2_ in homes with gas stoves and, to a lesser extent, in those with gas heating systems in 96 homes in Quebec City. Another study by Héroux et al. [21] identified the use of a gas stove as a significant predictor for increased NO_2_ levels in 145 homes in Regina. Both studies found no significant association between the use of gas appliances and indoor acetaldehyde levels. Previous research has provided valuable insights into the association between air pollutants and the presence of gas stoves and gas heating systems in homes. However, the impact of gas or oil combustion on indoor CO levels remained unexplored in these studies.

To expand our research across multiple regions and larger populations and to explore the association between fossil fuel combustion and indoor CO levels, we investigated the relationships between residential fossil fuel combustion and the levels of key air pollutants including NO_2_, CO, formaldehyde, and acetaldehyde in homes in Edmonton, Halifax, Windsor, and previously studied Regina. These studies provide a large and diverse sample of Canadian urban environments and housing characteristics. In addition to combustion sources, we examined other indoor and outdoor pollutant sources, along with household dynamics and occupant behaviors, to provide a comprehensive analysis of factors influencing IAQ. The results are expected to enhance our understanding of the relationship between indoor air quality and residential fossil fuel combustion in Canadian homes.

## Methods

### Study design

From 2005 to 2010, Health Canada and its collaborators conducted residential IAQ studies in four Canadian cities: Windsor (2005, 2006) [22], Regina (2007) [21], Halifax (2009) [23], and Edmonton (2010) [24]. The four cities are located in different provinces across Canada, with diverse climates. Figure S1 in the Supplemental Information (SI) shows the location of the cities. Halifax, the capital of Nova Scotia, has a humid continental climate, with warm summers and relatively mild winters due to its coastal location. Windsor, located in Southwestern Ontario, also has a humid continental climate but with hot, humid summers and cold winters, influenced by its proximity to the Great Lakes. Edmonton, the capital of Alberta, also features a humid continental climate with cold, dry winters and mild to warm summers. Regina, located in Saskatchewan, has a more extreme continental climate, characterized by very cold, dry winters and warm summers.

In Edmonton, Halifax, and Regina, homes were selected using stratified sampling according to the year of home construction. In Windsor, homes with asthmatic children aged 6–14 years were selected randomly, with a preference for households spatially distributed across the city. This selection was part of historical efforts to study respiratory health and environmental exposures [25], and is not expected to bias our current analysis of impacts of fossil fuel combustion and housing characteristics on indoor air quality. All homes were non-smoking homes. Although homes with smokers were recruited in Regina, they were excluded from the analysis to ensure comparability across the four cities. All IAQ studies were approved by the Health Canada Research Ethics Board, and informed consent was obtained from all participants.

### Measurements

All measurements were conducted by trained field technicians. A baseline questionnaire was administered by the technicians in the homes of each participant to collect information about housing characteristics. Additionally, a daily questionnaire was filled out by participants to record activities from the past 24 h that might affect IAQ, such as cooking, opening windows, and use of personal care products.

Air pollutants were measured over five consecutive days during winter and summer in Regina and Windsor and for seven days in Edmonton and Halifax. Indoor and outdoor 24-h integrated NO_2_ concentrations were measured daily using Ogawa Passive Samplers (Ogawa & Co., Pompano Beach, FL, USA). Indoor aldehydes were measured over 24 h each sampling day using UMEx 100 Passive Samplers (SKC Inc., Eighty Four, PA, USA). Indoor CO concentrations were monitored continuously at one-minute intervals with the Langan T15n CO Measurer (Langan, San Francisco, CA, USA). Aldehydes and CO were collected in Edmonton, Halifax, and Regina, but not in Windsor. Indoor temperature and relative humidity (RH) were continuously recorded using YES-206LH monitors (Yes Environment Technologies Inc., Delta, BC, Canada) in Edmonton and Halifax, and Smart Reader Plus 2 (ACR Systems Inc., Surrey, BC, Canada) in Windsor and Regina. Home air exchange rates were measured every 24 h using the perfluorocarbon tracer (PFT) technique [26], with PFT emitters placed at the four corners of the main floor, and a capillary absorption tube detector located in the center of the main floor. Indoor sampling equipment was positioned at breathing height (approximately 1.5 meters) in the living or family room, where participants typically spent most of their waking time. Outdoor samplers were placed in the backyard, ensuring placement away from combustion sources such as barbeques.

### Statistical analysis

The air pollutants studied included NO_2_, CO, formaldehyde, and acetaldehyde. The NO_2_ and aldehydes were measured daily, while CO was monitored continuously throughout the sampling period. In addition to calculating the daily average concentration of CO, we calculated the peak hourly CO levels to assess the short-term impact of the pollutant sources. The peak hourly CO was computed by taking the maximum value from the moving hourly average CO concentrations for each sampling day. For data below the detection limit, we used a robust regression on order statistics function from the NADA package in R to estimate non-detect values [27]. All pollutant concentrations followed a lognormal distribution, and the descriptive statistics were presented using the geometric mean (GM), the 95% confidence intervals (CIs) of the GM, the geometric coefficient of variation (GCV), and the interquartile range (IQR).

Our goal was to develop exploratory models to gain insights into complex data patterns, incorporating marginally significant variables (*p* < 0.1). This approach enabled us to identify variables that, while not reaching the 0.05 significance threshold, offered valuable insights into the relationships between pollutant concentrations and influential factors, which can inform further research.

To evaluate the influence of various factors on pollutant concentrations, we included the following into the analysis. The dependent variables were the natural logarithm of 24-h indoor concentrations of NO_2_, CO, formaldehyde, acetaldehyde, and the peak hourly CO concentrations. The independent variables included fossil fuel related sources (fuel types for cooking, main and supplemental heating, as well as water heating, presence of gas clothes dryers, and pilot lights on ovens or dryers), housing characteristics (dwelling type, year of construction, ventilation systems, presence of attached garages, air conditioning, range hoods, humidifiers, and dehumidifiers), demographic factors (homeowner’s racial and ethnic group, highest level of education, and household income), indoor environmental conditions (temperature, relative humidity (RH), and air exchange rate), outdoor pollutant concentration (as available), and occupant behaviors (cooking, burning candles, major renovations, painting, adding new furniture or rugs, use of personal care and cleaning products, and opening windows).

Most of the categorical variables were transformed into binary (yes/no) variables to improve interpretability, while some remained as categorical to show underlying patterns within those categories. Due to the limited sample size, the four homes that used propane for cooking were categorized under gas cooking. Additionally, we created composite variables by combining the fuel type of appliances with the home age category to evaluate whether the age of the home, potentially serving as an indicator of older appliances, influences pollutant emissions.

We conducted analyses of data from each city and also performed a pooled analysis combining data from all cities, for winter and summer. The city-specific analysis highlighted unique regional trends that might be overlooked in the combined data, while also validating the consistency of effects between cities. The pooled analysis increased the sample size, especially for specific binary variables such as the presence of gas clothes dryers. Furthermore, the pooled analysis not only enhanced the statistical power and generalizability of our findings but also helped to reduce city-specific biases, such as those influenced by dominant regional heating fuels.

In Regina homes, where NO_2_ and aldehydes were measured only on the first day of each sampling season, we used general linear models for analysis. For NO_2_ and aldehydes in other cities, as well as CO in all cities, which were measured repeatedly in each home, we employed a linear mixed model with a first-order autoregressive (AR1) covariance structure to account for both fixed and random effects. In the city-specific analyses, home was treated as a random effect to account for variation between homes. For the pooled analysis of NO_2_ and aldehydes, we averaged daily concentrations from each home in Halifax, Edmonton, and Windsor to align with Regina’s single measurement approach. In this analysis, city was treated as a random effect to account for variations between cities. For the pooled analysis of CO, where repeated measurements were taken in all homes, we included both the city and home as random effects to account for variations at both levels.

To select candidate variables for multivariate analysis, we used univariate mixed model analysis, employing F-tests to assess the association between each individual factor and pollutant levels. Variables with a *p*-value less than 0.25 were considered. Additionally, those that were non-significant but had theoretical relevance based on prior knowledge and subject-matter expertise were also included. We assessed multicollinearity among the candidate variables using variance inflation factors and retained the variable with a stronger association with the dependent variable in cases of high collinearity. Using backward elimination, we removed non-significant variables (*p* ≥ 0.1) and those with counterintuitive relationships relative to expected pollutant source directions. After this elimination, we assessed the preliminary model to determine if any excluded variables merited re-inclusion. Residual and influence analysis, including Cook’s Distance and restricted likelihood distance, was performed to examine the assumptions of linearity and homoscedasticity and to identify potential outliers or influential observations that might affect the model’s estimates. As variable selection can be sensitive to changes in the data, we repeated the above analysis procedures if any variables or influential points were eliminated. The final model decision was based on Akaike’s Information Criterion, the significance of the variables, and subject-matter knowledge.

For reporting the results, we calculated percent changes by applying exponential transformations to the beta coefficients (percent change = (exp (β) − 1) × 100%). Percent changes provide a more interpretable and meaningful perspective on the magnitude and direction of the associations for log-transformed models. To provide a detailed view of the exposure levels and enable a direct comparison of average pollutant concentrations across different groups defined by categorical variables in the model, we reported the GM values of pollutants and their 95% CIs for each variable in the SI. All the analyses were conducted using SAS Enterprise Guide 7.1 (SAS Institute Inc.) and R 4.1.1 (R Core Team, 2021).

## Results

### Household characteristics

Air pollutant samples were collected from a total of 344 homes in four cities: Edmonton (22%), Halifax (17%), Regina (35%), and Windsor (26%). An overview of the characteristics of these homes is presented in Table 1. The majority of the residences were owner-occupied detached houses (92%) and built before 1990 (69%).

**Table 1:** General characteristics and energy preferences of the 344 sampled homes.

Home characteristic		Number of homes				
		Edmonton	Halifax	Regina	Windsor	Pooled
Number of homes		74	58	121	91	344
Dwelling type	Detached house	69	53	111	84	317
	Townhouse, duplex, or triplex	5	2	4	6	17
	Other	0	3	6	1	10
Construction year	Before 1949	15	17	11	16	59
	1949–1989	31	23	79	47	180
	1990 or after	28	18	29	24	99
	Unknown	0	0	2	4	6
Home size (m^2^)	<93	1	1	6	7	15
	93–186	13	22	42	34	111
	186–279	35	23	46	34	138
	≥279	20	11	26	13	70
	Unknown	5	1	1	3	10
Type of garage	Detached or separate carport	43	10	42	21	116
	Attached	28	25	58	39	150
	No garage	0	23	21	30	74
	Unknown	3	0	0	1	4
Main heating fuel	Electricity	0	12	5	6	23
	Gas or propane	74	6	110	27	217
	Oil	0	33	1	0	34
	Wood	0	1	0	1	2
	Other or mixed	0	0	5	0	5
	Unknown	0	6	0	57	63
Supplemental heating	Gas fireplace	22	11	34	18	85
	Gas space heater	1	0	0	0	1
	Wood heater (fireplace, stove)	5	21	17	4	47
	Electric space heater	22	13	19	18	72
	Kerosene space heater	0	0	1	0	1
	Other	4	4	8	27	43
	None	32	12	52	27	123
Water heating fuel	Electricity	0	17	12	5	34
	Gas or propane	73	5	108	82	268
	Oil	0	34	0	0	34
	Wood	1	0	0	0	1
	Other	0	2	1	0	3
	Unknown	0	0	0	4	4
Cooking fuel	Electricity	58	46	83	67	254
	Gas	15	9	37	23	84
	Propane	1	3	0	0	4
	Other	0	0	1	1	2
Presence of range hoods	Yes	51	50	78	29	208
	No	23	8	43	13	87
	Unknown	0	0	0	49	49
Presence of gas clothes dryers	Yes	6	7	0	7	20
	No	68	51	0	84	203
	Unknown	0	0	121	0	121
Presence of pilot lights on ovens or clothes dryers	Yes	2	1	9	11	23
	No	72	57	112	80	321

Natural gas was the most popular source of energy for home heating and water heating, in 63% and 78% of homes, respectively. Oil heating was most prevalent in Halifax, used in 57% of homes, but its usage in other cities was marginal (0.3% on average). Electric heating was less common, accounting for 7% of home heating and 10% of water heating. For supplemental heating purposes, 25% of homes used gas fireplaces, and 0.3% used gas space heaters.

Compared to its use for heating, gas was less commonly used for large appliances. For cooking fuel, electricity was the predominant choice (74% of homes), while gas was used by 24% of households and propane by only 1%. About 6% of homes had a gas clothes dryer, and 7% had a continuously burning pilot light on ovens or clothes dryers.

### Air pollutant concentrations and influencing factors

The overall GM concentrations across all cities and seasons for the pollutants were: NO_2_ of 9.0 µg/m^3^, daily CO of 1.7 ppm, peak hourly CO of 2.1 ppm, formaldehyde of 24.0 µg/m^3^, and acetaldehyde of 7.5 µg/m^3^. Descriptive statistics for pollutant concentrations by city and season are presented in Table 2.

**Table 2:** Summary of pollutant concentrations by city and season, including geometric mean (GM) and 95% confidence intervals (CIs) of GM, geometric coefficient of variation (GCV), and interquartile range (IQR).

Pollutant	City	Season	*N* (homes)	BDL^a^ (%)	GM (GCV), 95% CIs of GM	IQR
NO_2_ (µg/m^3^)	Edmonton	Summer	50	9	10.71 (0.79), 9.95–11.54	7.39–16.04
		Winter	50	2	14.27 (0.77), 13.28–15.34	9.22–21.80
	Halifax	Summer	50	43	4.3 (1.29), 3.86–4.78	2.17–7.79
		Winter	50	14	6.52 (1.36), 5.83–7.30	3.41–13.75
	Regina^b^	Summer	93	0	8.2 (0.8), 7.09–9.48	5.41–14.18
	Windsor	Summer	86	6	8.55 (4.23), 7.25–10.09	6.89–19.81
		Winter	87	2	13.24 (2.00), 11.72–14.95	9.52–24.81
Peak hourly CO (ppm)	Edmonton	Summer	47	-	3.41 (0.41), 3.27–3.56	2.79–4.17
		Winter	47	-	2.42 (0.56), 2.29–2.56	1.77–3.11
	Halifax	Summer	42	-	1.27 (0.89), 1.16–1.38	0.71–2.54
		Winter	42	-	2.56 (0.85), 2.35–2.80	1.53–3.63
	Regina	Summer	92	-	1.85 (0.48), 1.77–1.93	1.46–2.33
		Winter	82	-	1.94 (1.13), 1.77–2.13	1.57–3.10
Daily CO (ppm)	Edmonton	Summer	47	-	2.70 (0.39), 2.59–2.81	2.35–3.35
		Winter	47	-	1.75 (0.40), 1.67–1.82	1.36–2.24
	Halifax	Summer	42	-	1.04 (0.97), 0.94–1.14	0.58–2.23
		Winter	42	-	1.91 (0.87), 1.75–2.09	1.29–2.90
	Regina	Summer	92	-	1.48 (0.43), 1.43–1.54	1.22–2.00
		Winter	82	-	1.50 (1.04), 1.37–1.63	1.34–2.39
Formaldehyde (µg/m^3^)	Edmonton	Summer	50	0	24.76 (0.81), 22.94–26.73	15.30–40.43
		Winter	50	0	21.18 (0.52), 20.10–22.31	16.67–28.81
	Halifax	Summer	50	0	25.48 (0.81), 23.61–27.49	15.46–39.43
		Winter	33	0	22.52 (0.81), 20.46–24.78	12.78–36.33
	Regina	Summer	93	0	32.09 (0.61), 28.56–36.05	23.19–47.11
		Winter	83	0	23.83 (0.48), 21.57–26.33	18.73–33.65
Acetaldehyde (µg/m^3^)	Edmonton	Summer	50	2	8.10 (0.85), 7.48–8.77	5.07–13.90
		Winter	50	0	8.89 (0.54), 8.43–9.38	6.45–12.08
	Halifax	Summer	50	0	5.97 (0.81), 5.53–6.44	3.80–9.34
		Winter	33	8	6.04 (1.11), 5.35–6.81	3.54–11.40
	Regina	Summer	93	1	9.68 (0.82), 8.35–11.22	6.78–15.86
		Winter	83	1	8.82 (0.75), 7.61–10.21	5.76–14.51

^a^BDL: below the detection limit.

^b^Regina winter NO_2_ data were invalid and therefore not presented or analyzed [21].

Seasonal variations in NO_2_ levels were significant across all cities, with higher concentrations in winter, as determined by mixed-effects models with *F*-tests. CO levels were higher in winter than summer in Halifax, Regina, and Windsor, while Edmonton showed the opposite trend. No significant seasonal variations were found for formaldehyde and acetaldehyde. A weak positive correlation (*r* = 0.32) was noted between formaldehyde and acetaldehyde concentrations.

Table 3 presents the associations between pollutant concentrations and influencing factors by city and in the pooled analysis for summer and winter. The GM values of pollutant concentrations across various categorical groups and per unit change in continuous variables are presented in the SI, Table S1. All variables presented in Tables 3, 4, Table S1, and the description below are statistically significant (*p* < 0.05) unless indicated as marginally significant (0.05 ≤ *p* < 0.1).

**Table 3:** Associations between residential fossil fuel combustion and other factors and indoor air pollutant concentrations by city and in a pooled analysis.

Pollutant	Season	Factor	Percent change in pollutant concentrations and 95% confidence intervals				
			Edmonton	Halifax	Regina	Windsor	Pooled
NO_2_ (µg/m^3^)	Summer	Presence of gas stoves (yes)	31 (−2 to 75)^†^	181 (89 to 319)****	125 (75 to 190)****	138 (87 to 204)****	114 (77 to 159)****
		Presence of gas clothes dryers (yes)					47 (10 to 97)*
		Outdoor NO_2_ concentration (per 10% increase)	4 (3 to 4)****	5 (4 to 6)****	2 (0.6 to 3)**	8 (7 to 8)****	7 (6 to 8)****
		Home construction year					
		─ after 1990 (reference)					
		─ 1949 to 1990	6 (−17 to 35)	−18 (−42 to 15)		38 (7 to 77)*	13 (−6 to 35)
		─ before 1949	58 (16 to 116)**	44 (−2 to 110)^†^		32 (−4 to 82)^†^	31 (5 to 64)*
		Windows opened (yes)	31 (11 to 55)**		30 (1 to 68)*	53 (29 to 83)****	36 (2 to 81)*
		Indoor temperature (°C)		7 (2 to 12)**	8 (2 to 15)**		
		Indoor relative humidity (%)			2 (−0.2 to 4)^†^		
	Winter	Presence of gas stoves (yes)	103 (60 to 157)****	354 (168 to 668)****		202 (64 to 458)**	191 (122 to 279)****
		Heating system fuel type					
		─ electric (reference)					
		─ gas homes built before 1949	65 (29 to 111)***	38 (−48 to 267)		66 (−36 to 328)	62 (−2 to 168)^†^
		─ gas homes built 1949 to 1990	14 (−9 to 42)			15 (−43 to 133)	30 (−19 to 109)
		─ gas homes built after 1990^a^		34 (−57 to 320)		−25 (−74 to 118)	11 (−35 to 89)
		─ oil		85 (17 to 192)**			58 (4 to 141)*
		Presence of gas clothes dryers (yes)					54 (3 to 129)*
		Candles used (yes)	22 (8 to 37)**	28 (8 to 51)**			53 (22 to 93)***
		Outdoor NO_2_ concentration (per 10% increase)	3 (2 to 3)****	2 (1 to 3)****		3 (0.7 to 5)*	4 (1 to 6)**
		Close to construction sources (yes)		75 (15 to 166)*			40 (11 to 76)**
		Indoor temperature (°C)	4 (−0.1 to 9)^†^	9 (1 to 17)*			
		Indoor relative humidity (%)		4 (1 to 7)**			
Peak hourly CO (ppm)	Summer	Presence of gas stoves (yes)			26 (7 to 49)**		
		Candles used (yes)		12 (1 to 25)*			
		Windows opened (yes)	−6 (−12 to 0)*				
		Indoor temperature (°C)	8 (7 to 10)****	3 (0 to 5)^†^	3 (0.7 to 5)**		5 (4 to 7)****
		Indoor relative humidity (%)	2 (1 to 3)****	−1 (−2 to −0.1)*			
		Air exchange rate (h^-1^)		−17 (−24 to −9)****			−5 (−10 to −0.1)*
	Winter	Presence of gas stoves (yes)	55 (12 to 114)**				43 (2 to 102)*
		Indoor temperature (°C)	11 (6 to 16)****	6 (−0.6 to 13)^†^			4 (0.9 to 7)**
Daily average CO (ppm)	Summer	Presence of attached garages (yes)			19 (1 to 39)*		
		Indoor relative humidity (%)	1 (0.5 to 2)***	−0.7 (−1 to −0.1)*			
		Indoor temperature (°C)	7 (6 to 8)****	2 (−0.1 to 4)*	3 (2 to 5)****		5 (3 to 6)****
		Air exchange rate (h^-1^)	−9 (−14 to −5)***				−5 (−8 to −2)**
	Winter	Presence of attached garages (yes)	19 (−1 to 43)^†^				
		Indoor relative humidity (%)					0.8 (−0.1 to 2)^†^
		Indoor temperature (°C)	6 (3 to 10)***				3 (0.8 to 5)**
Formaldehyde (µg/m^3^)	Summer	Home construction year					
		─ after 1990 (reference)					
		─ 1949 to 1990	−20 (−38 to 3)^†^	−41 (−57 to −19)**	−24 (−41 to −3)*		−33 (−44 to −19)****
		─ before 1949	−51 (−65 to −31)***	−47 (−63 to −23)**	−18 (−45 to 24)		−48 (−59 to −34)****
		Major renovations within the last month (yes)		55 (3 to 134)*	65 (−0.9 to 174)^†^		83 (24 to 170)**
		Hair spray used (yes)		11 (1 to 21)*			
		Windows opened (yes)	−8 (−14 to −3)**	−15 (−23 to −6)**	−27 (−42 to −7)**		−28 (−47 to −2)*
		Air cleaner used (yes)			−47 (−63 to −22)**		
		Presence of dehumidifiers (yes)		−30 (−47 to −7)*			−28 (−43 to −9)**
		Air conditioning used (yes)	−10 (−20 to 1)^†^		29 (4 to 60)*		
		Indoor relative humidity (%)	2 (1 to 3)****	1 (0.4 to 2)**			
		Indoor temperature (°C)	8 (6 to 10)****	12 (9 to 14)****			6 (1 to 10)*
		Air exchange rate (h^-1^)	−44 (−48 to −40)****	−41 (−44 to −37)****			−51 (−59 to −42)****
	Winter	Home construction year					
		─ after 1990 (reference)					
		─ 1949 to 1990	−13 (−30 to 9)	−47 (−63 to −24)**	−30 (−42 to −15)***		−27 (−38 to −15)****
		─ before 1949	−38 (−52 to −20)***	−55 (−69 to −35)***	−54 (−65 to −40)****		−46 (−55 to −34)****
		Indoor relative humidity (%)	3 (2 to 4)****	5 (4 to 6)****	4 (3 to 5)****		4 (2 to 5)****
		Indoor temperature (°C)	9 (7 to 12)****	10 (8 to 13)****	10 (6 to 14)****		8 (4 to 11)****
		Air exchange rate (h^-1^)	−23 (−37 to −7)**	−35 (−53 to −10)*			−29 (−50 to 2)^†^
Acetaldehyde (µg/m^3^)	Summer	Home construction year					
		─ after 1990 (reference)					
		─ 1949 to 1990	−2 (−37 to 52)	−16 (−47 to 32)			−10 (−34 to 22)
		─ before 1949	−41 (−67 to 4)^†^	−37 (−63 to 5)^†^			−39 (−58 to −12)**
		Perfume used (yes)			23 (−3 to 57)^†^		
		Air conditioning used (yes)			73 (36 to 119)****		
		Windows opened (yes)			−36 (−50 to −17)***		
		Air cleaner used (yes)			−40 (−62 to −6)*		
		Air exchange rate (h^-1^)	−12 (−22 to 0.4)^†^	−11 (−18 to −4)**			−13 (−19 to −6)***
	Winter	Home construction year					
		─ after 1990 (reference)					
		─ 1949 to 1990	14 (−18 to 57)	−59 (−78 to −23)**	−35 (−54 to −9)*		−23 (−38 to −4)*
		─ before 1949	−27 (−50 to 6)^†^	−61 (−79 to −26)**	−47 (−68 to −14)*		−34 (−50 to −14)**
		Cooked with oil (yes)			31 (0.7 to 70)*		
		Indoor relative humidity (%)		3 (0.9 to 5)**	5 (3 to 7)****		4 (2 to 6)****
		Indoor temperature (°C)			10 (3 to 17)**		6 (0.9 to 10)*
		Air exchange rate (h^-1^)	−22 (−41 to 3)^†^				−58 (−74 to −31)***

Results are expressed as percent changes with 95% confidence intervals in pollutant concentrations, corresponding to per unit changes in continuous variables or to different categories of categorical variables, evaluated using mixed models and general linear models. Statistical significance is denoted at *p* < 0.1, with specific levels indicated as †*p* < 0.1, *p < 0.05, **p < 0.01, ***p < 0.001, ****p < 0.0001.

^a^In Edmonton, all homes in the study used gas heating; therefore, ‘homes built after 1990’ was used as a reference category for heating system fuel type in the Edmonton winter model.

The presence of gas stoves was significantly associated with increased indoor NO₂ and the peak hourly CO concentrations, with a larger impact in winter than in summer. The pooled analysis showed a 191% increase in NO₂ (GM of 29.8 µg/m^3^) and a 43% increase in the peak hourly CO (GM of 2.92 ppm) during winter. In comparison, the increase in summer was 114% for NO₂ (GM of 17.2 µg/m^3^), and the increase for peak hourly CO was not statistically significant. The IAQ levels associated with gas stoves did not show significant variation across homes of different age categories.

In addition to assessing the overall association between gas stove use in homes and pollutant concentrations, we further examined the associations between cooking duration and the increase in indoor NO₂ and peak hourly CO levels in homes with gas stoves. The corresponding percentage increase in NO₂ and peak hourly CO concentrations for every 10-min increment of gas stove use is shown in Table 4. It is important to note that due to a lack of data availability, these estimates did not account for variations in pollutant emissions due to factors such as the number of burners used or the heat settings during gas cooking.

**Table 4:** Associations between cooking duration and indoor NO_2_ and peak hourly CO levels in homes with gas stoves.

Pollutant	Season	Factor	Percent change in pollutant concentrations and 95% confidence intervals		
			Edmonton	Halifax	Pooled^a^
NO_2_ (µg/m^3^)	Summer	Duration of gas stove use (per 10 min)		6 (−0.1 to 12)^†^	5 (0 to 11)*
	Winter	Duration of gas stove use (per 10 min)	2 (0.9 to 4)**	5 (0.4 to 11)*	1 (0 to 2)*
		Outdoor NO_2_ concentrations (per 10% increase)	3 (2 to 5)***		
Peak hourly CO (ppm)	Winter	Duration of gas stove use (per 10 min)			2 (0.4 to 4)*

Results are presented as the percent change and 95% confidence intervals in pollutant concentrations associated with per unit changes in continuous variables, evaluated using mixed models and general linear models. Statistical significance is denoted at *p* < 0.1, with specific levels indicated as ^†^*p* < 0.1, *p < 0.05, **p < 0.01, ***p < 0.0001.

^a^In Regina, despite the lack of significant associations between cooking duration and both indoor NO_2_ and peak hourly CO levels in homes with gas stoves, these homes were still included in the pooled analysis.

In winter, gas heating in older homes (built before 1949) was associated with a 62% increase in NO_2_ levels in the pooled analysis (GM of 21.9 µg/m^3^), with marginal significance (*p* = 0.057). Oil heating was only presented in Halifax homes, and was associated with an 85% increase in NO₂ in Halifax (GM of 14.4 µg/m^3^) and a 58% increase in the pooled analysis (GM of 21.3 µg/m^3^), both statistically significant. For homes using oil or electric heating, indoor NO_2_ levels did not significantly vary across different home ages. Additionally, the pooled analysis indicated a 47% increase in NO₂ levels in summer (GM of 14.2 µg/m^3^) and 54% in winter (GM of 21.7 µg/m^3^) associated with gas clothes dryers. We did not conduct a city-level assessment of the association between gas clothes dryers and pollutant concentrations due to the small sample size in each city.

Occupant activities, such as the burning of candles, were associated with increased NO_2_ (GM of 21.7 µg/m^3^ in pooled analysis) and peak hourly CO (GM of 1.42 ppm in Halifax). Aldehyde levels were associated with recent major renovations (83% increase in formaldehyde in the pooled analysis, GM of 46.2 µg/m^3^), cooking with oil (31% increase in acetaldehyde in Regina, GM of 10.6 µg/m^3^), and the use of personal products, including hair spray (11% increase in formaldehyde in Halifax, GM of 35.6 µg/m^3^) and perfume (23% increase in acetaldehyde in Regina with marginal significance, GM of 10.0 µg/m^3^). The use of air cleaners was significantly associated with reduced formaldehyde (47% reduction, GM of 25.0 µg/m^3^) and acetaldehyde (40% reduction, GM of 6.99 µg/m^3^) levels in Regina. The presence of dehumidifiers was significantly associated with reduced formaldehyde levels in pooled analysis (28% reduction, GM of 29.0 µg/m^3^) in summer.

A range of housing characteristics were associated with changes in pollutant levels. Having attached garages was associated with a 19% increase in daily CO levels in Regina (GM of 2.02 ppm), and marginally significant increases of 19% in Edmonton (GM of 1.62 ppm). Older homes, especially those built before 1949, showed 34–48% lower levels of aldehyde in the pooled analysis. However, these older homes also showed significantly higher levels of NO_2_ in summer, with a 31% increase in the pooled analysis (GM of 13.5 µg/m^3^).

For ventilation-related parameters, higher air exchange rates and opening of windows were associated with reductions in the peak hourly CO and aldehyde concentrations. Outdoor NO₂ levels were significant contributors to indoor sources, particularly in summer, with a 7% increase in indoor NO₂ for every 10% rise in outdoor NO₂ compared to a 4% increase in winter, as shown in the pooled analysis. Additionally, homes located near construction sources had higher indoor NO_2_ levels, with a 40% increase in the pooled analysis.

## Discussion

### Pollutant levels and prevalence of fossil fuel use in homes

Pollutant concentrations in the studied homes were generally comparable to other studies conducted in Canada. The geometric mean (GM) concentration of NO_2_ was 9.0 ug/m^3^, comparable to the measurements in Quebec City with a GM of 8.3 µg/m^3^ [20], and a mean of 7 ppb (equivalent to 13 ug/m^3^) [28]. However, it was much lower than the mean indoor level of 14 ppb (equivalent to 26 ug/m^3^) reported in Hamilton [29]. In our study, the GM for daily CO concentrations in homes was 1.6 ppm. In Quebec City homes, CO concentrations were mostly undetectable (<1 ppm), with occasional higher levels found mainly in basements with wood stoves [28]. Our formaldehyde concentrations, with a GM of 24 µg/m³, fell within the range reported for other regions in Canada, including Prince Edward Island (median 33.2 µg/m³), Quebec City (GM 29.5 µg/m³), and the remote First Nations Communities in northwestern Ontario (GM of 16 µg/m³) [20, 30, 31]. Lastly, our acetaldehyde GM level of 7.6 µg/m³ was lower than in Prince Edward Island (20.2 µg/m³) [30].

The percentage of studied homes with gas stoves (24%) was higher than the national surveys, which reported gas stove usage in the range of 14–16% [12, 13]. The discrepancy may be explained by our intentional decision to include homes with gas stoves to ensure adequate representation, especially in light of the unavailability of certain national surveys during the study period. The prevalence of gas use for home and water heating, as well as the regional variations in the studied homes, were in alignment with the national census data [1].

### Association between fossil fuel combustion and indoor air quality

The presence of gas stoves was strongly associated with increased indoor NO_2_ concentrations in studied homes. This was consistent across all cities, showing a more pronounced increase in winter compared to summer. The positive correlation between gas stove usage and elevated indoor NO_2_ concentrations aligned with findings reported in previous research [3, 6, 10, 32–34]. The larger increase observed in winter may be related to reduced ventilation and increased cooking activities. Research examining cooking patterns in Canadian homes indicated that cooking frequency tends to be higher in winter compared to summer [12, 35]. This seasonal variation in cooking behavior, combined with reduced ventilation, likely amplifies the effect of gas stoves on indoor NO_2_ levels during winter months.

Homes with gas stoves also had increased peak hourly CO levels, with a greater increase in winter than in summer, paralleling the trend observed with NO_2_. While gas cooking was significantly associated with short-term CO levels, it was not associated with the daily average CO concentrations. This suggests that short-term fluctuations in CO levels, possibly linked to specific activities such as cooking on gas stoves, might not be reflected in daily average measurements. Elevated indoor CO levels associated with gas stove cooking have been reported by several studies [2, 32]. However, it is important to note that other studies have observed only minor or negligible increases in CO levels from gas stove use [5, 36]. These varying results suggest that the association between gas stoves and CO levels is influenced by many factors, such as the efficiency of gas combustion, the duration of stove use, ventilation conditions, and specific housing characteristics.

Many studies have shown that the use of vented range hoods during cooking can significantly reduce exposure to air pollutants generated from cooking [3, 5, 37]. Our study did not observe a correlation between range hood use and lower pollutant concentrations. Despite the fact that 71% of homes had a range hood (only 39% vented to outside), their usage during cooking was only 18% (17% with gas stove cooking). This low use rate was consistent with the findings of a national survey [12], where only about one-third of the survey respondents reported regularly using their range hoods, and many perceived them as ineffective. For cooking methods that produce little smoke or odor, such as boiling or steaming, people may not perceive the need to turn on their range hoods. Enhancing public awareness of emissions from gas burners and their associated health risks is important for encouraging more frequent use of range hoods to reduce exposure to air pollutants.

In our study, increased ventilation was associated with lower indoor levels of CO, formaldehyde, and acetaldehyde, whereas the positive association between window opening and indoor NO₂ levels likely reflects contributions from outdoor sources. Outdoor NO_2_ sources, such as road transportation, have been reported in many studies [19, 38–40]. In our study, homes located near construction activities had higher indoor NO₂ levels, indicating that heavy diesel traffic and the operation of construction equipment nearby may be potential contributors. This suggests that specific pollutants, their sources (both indoor and outdoor), and home conditions are important considerations in ventilation practices to effectively manage IAQ. For homes with gas stoves, indoor NO_2_ concentrations associated with their use (GM of 17.2 µg/m³ in summer and 29.8 µg/m³ in winter, as shown in Table S1) were estimated to be higher than outdoor levels (GM of 8.4 µg/m³ in summer and 23.7 µg/m³ in winter), suggesting that opening windows could help dilute indoor NO_2_. However, this effect was not sufficient to alter the overall positive association between window opening and indoor NO₂ levels, which was primarily driven by the majority of homes without significant indoor sources.

Gas heating was associated with a 62% increase in winter NO_2_ levels in older homes (built before 1949), with marginal significance in pooled analysis. There have been fewer studies investigating the IAQ impact from central gas or oil heating systems (furnaces or boilers) than gas stoves and unvented gas heaters. In the Gilbert et al. [20] study of Quebec City homes, the gas heating system was significantly associated with elevated indoor NO_2_ levels, showing a 54% increase. The association of oil heating in Quebec City homes was marginally significant, with a 22% increase in NO_2_. The reason for the observed association between NO_2_ and gas or oil heating systems was not well understood. Compared to unvented gas stoves, gas or oil heating systems are expected to have a minimal impact on IAQ as they are vented, as required by the National Building Code of Canada and ASHRAE standard 62.2. One possible reason could be backdrafting or combustion spillage, mainly resulting from house depressurization [41–44]. Older homes are more likely equipped with natural draft furnaces and boilers, which are particularly susceptible to these issues. While the assessment of combustion spillage and backdrafting commonly involves monitoring CO and CO_2_ concentrations, the measurements of NO_2_ in these tests are relatively uncommon and require further investigations [43, 44]. Another possible reason for elevated combustion emissions could be improperly installed vent systems or undersized connectors and vents [41, 42]. In addition, research showed that maintenance of gas appliances is important to ensure proper airflow and ventilation. Combustion equipment that is well-tuned and regularly maintained is likely to emit fewer pollutants and vent more efficiently [41, 42, 44]. In the absence of specific details about furnaces or boilers, such as information on venting types (natural draft, induced draft with an exhaust fan, or direct venting with sealed combustion), device age, and maintenance frequency, we used the age of the homes as a proxy to estimate variations in heating device parameters. In older homes, there is a greater likelihood of having naturally drafted heating systems or devices that are inadequately maintained, leading to elevated NO_2_ levels. The lack of a clear relationship between home age and NO_2_ emissions from oil heating might be attributable to a small and unevenly distributed sample size across home age categories, potentially reducing the statistical power to detect a pattern, if one exists, or it might suggest that there is no underlying pattern. More research is needed to confirm our findings and explain the association.

We did not observe a strong association between fossil fuel combustion and indoor aldehyde levels. Several studies have explored aldehyde emissions, with some reporting increased formaldehyde levels from gas cooking burners and ovens, tankless water heaters, and furnaces [2, 4, 7, 14]. The emissions varied depending on gas quality and cooking methods [4, 7]. For example, simmering on a gas burner without local exhaust ventilation significantly increased formaldehyde levels to a greater extent than when burners were operated at a high setting [4]. Conversely, other studies have reported no associations between the use of gas appliances and indoor aldehyde levels [3, 20, 21, 32]. The discrepancies in findings could be attributed to differences in sampling methods (integrated, continuous), burner operations (flame temperature, number of burners in use), ventilation conditions, and other indoor sources such as building materials.

### Association between other factors and indoor air quality

Housing characteristics, including the presence of attached garages and the age of the home, were significantly associated with IAQ parameters. The presence of attached garages was associated with higher daily CO concentrations, consistent with studies that have investigated the infiltration of CO and other vehicular emissions from attached garages into homes [45–47]. Homes built before 1949 showed higher levels of NO₂ in summer. The results were marginally significant in the pooled analysis. Considering that the effect of gas stoves did not vary significantly across homes of various ages, the increase was unlikely due to older gas appliances. This might be attributed to older homes’ leakier nature, allowing more outdoor NO₂ infiltration [48, 49].

Older homes had lower levels of aldehydes compared with newer homes, potentially due to off-gassing from building materials. In terms of renovation activities, we examined the influence over different time frames, including the past one month, three months, six months, and one year. We observed that only renovations completed in the last month had a significant association with increased formaldehyde levels. This finding suggests that while renovations may cause a short-term increase in indoor formaldehyde levels, newer homes might consistently exhibit higher baseline levels of aldehydes, attributed to the continued off-gassing from building materials, in contrast to older homes where these emissions have decreased over the years. The elevation of indoor aldehyde levels due to off-gassing sources has been widely reported in studies conducted in Canada [20, 21, 30], and other countries [32, 50–52].

Burning candles was significantly associated with higher indoor NO_2_ and peak hourly CO. Previous research has identified a range of pollutants emitted from candle burning, including NO_x_, CO, particulate matter, and volatile organic compounds (VOCs), and characterized pollutant emission rates [53–55]. Specifically, Lee and Wang’s study [55] highlighted that NO_x_ was the most abundant pollutant emitted from candles among the air pollutants evaluated.

Higher indoor temperature and RH levels were associated with increased aldehyde levels. The findings are consistent with many studies that have shown elevated temperature and humidity in indoor environments can increase the emission rate of formaldehyde from building materials and furnishings [56–60]. The presence of dehumidifiers was significantly associated with reduced formaldehyde concentrations in summer. Dehumidifiers are recommended as one of the tools to reduce formaldehyde emissions by controlling or reducing humidity in homes; for example, by keeping RH below 50% in summer [61–63]. Our study found no significant difference in average RH levels between homes with and without dehumidifiers. However, homes with dehumidifiers had a lower maximum RH of 57% and showed less fluctuation in RH, with a range of 27% between the maximum and minimum RH values and an interquartile range (IQR) of 7%, compared to homes without dehumidifiers (maximum RH of 65%, range of 40%, IQR of 11%). The results suggest that dehumidifiers help to prevent high RH levels, where the effect of RH on formaldehyde emission rates becomes more significant [64].

Our study suggests potential associations between the use of hair sprays and perfumes and elevated aldehyde levels. Personal care products, especially those that are fragranced, can release a variety of organic compounds, including aldehydes [65, 66]. In our study, 53% of participants used hair sprays, and 56% used perfumes at least once during the sampling period. A recent U.S. survey indicated a significant lack of public awareness, with over two-thirds of respondents unaware of the air pollutant emissions from fragranced consumer products [67]. This suggests a need for public education on the potential risks associated with emissions from personal care products, which are often present in the breathing zone of users.

### Study limitations and strengths

This study has several limitations. The selection of homes was not random, with inclusion criteria such as owner-occupied, non-smoking homes, or those with asthmatic children (in Windsor). The study did not include apartment buildings, where the use of gas appliances may have a larger impact on occupants’ exposure due to factors such as smaller living spaces compared to detached houses or townhomes. All participants were homeowners. Rental homes, which may potentially be lower-income households, were not included. Rural areas, which often rely on propane, oil, and wood as non-electric energy sources due to limited access to gas lines, were not investigated. While the overall sample size of 344 homes was substantial for general analyses, small sample sizes for certain subgroups (e.g., homes with oil heating across various home age categories, homes using propane) may limit the statistical power to detect patterns. The accuracy of the self-reported questionnaire data was not evaluated. Due to logistical constraints, NO₂ and aldehydes in Regina were measured only on the first day of each sampling season. This single-day measurement was subject to random error and may not accurately represent typical exposure levels. Outdoor samples were only collected for NO₂ due to resource restrictions, limiting our ability to fully assess the influence of outdoor sources on indoor levels of CO and aldehydes. Additionally, the analysis used stove fuel type to represent homes’ cooking fuel. Although stoves and ovens are commonly integrated into a single unit and use the same fuel type, there might be scenarios where homes use different fuels for each, which may not be accurately reflected. Future research could focus on evaluating IAQ in apartment buildings and rural areas to enhance understanding of fossil fuel combustion exposure in these specific indoor environments.

A key strength of this study is its comprehensive scope, evaluating multiple air pollutants across winter and summer seasons and taking into account a variety of household dynamics and occupancy behaviors. By including homes from four Canadian cities, the study captures a wide range of climatic conditions and energy use patterns. Despite the data being collected over different years and in different cities, the use of consistent sampling methods ensures the comparability of results across these diverse settings and allows for combined city analyses. Among the pollutants we analyzed, CO exposure levels and their influential factors were investigated in Canadian homes for the first time, filling a gap in existing research.

### Practical implications

This study enhances the understanding of environmental exposure related to residential fossil fuel combustion in predominantly detached homes, which accounted for 52.6% of Canadian dwellings based on 2021 Census data [68]. The findings highlight differences in pollutant levels between homes with gas and electric cooking, which can inform advice on cooking practices to reduce emissions in homes. Transitioning to electric cooking appliances may help to significantly lower indoor NO₂ levels [69, 70]. Ensuring adequate ventilation through properly installed and maintained range hoods or other ventilation systems is crucial for all types of cooking to minimize exposure to cooking-generated air pollutants. The lack of association between range hood use and reduced pollutant levels highlights the importance of raising public awareness about emissions from gas burners and promoting more frequent use of range hoods. Furthermore, our findings suggest that specific pollutants, their sources (both indoor and outdoor), and home conditions are important considerations in ventilation practices to effectively manage IAQ.

## Conclusions

This study examined the associations between fossil fuel combustion and indoor air quality in 344 homes across Edmonton, Halifax, Regina, and Windsor. We found that the presence of gas stoves or gas clothes dryers was significantly associated with increased indoor NO_2_ concentrations, showing a greater increase in winter than in summer. Oil heating significantly elevated indoor NO_2_ levels in winter, while gas heating showed a marginal association with higher NO_2_ levels in older homes in the study (built before 1949). Peak hourly CO concentrations were strongly associated with the presence of gas stoves but not with other gas or oil appliances. Moreover, residential fossil fuel combustion was not significantly associated with levels of daily average CO, formaldehyde, and acetaldehyde in homes.

## Supplementary information

Supplementary information

## Data Availability

The datasets generated and/or analyzed during the current study are not publicly available due to consent agreements from the IAQ studies that restrict the sharing of raw data collected from participants.
