# Supplementary material for: Associations between residential fossil fuel combustion and indoor concentrations of nitrogen dioxide, carbon monoxide, and aldehydes in Canadian homes
Source: J Expo Sci Environ Epidemiol. 2025 Mar 14;35(4):649–60. doi: 10.1038/s41370-025-00762-6 (PMC12234359; doi:10.1038/s41370-025-00762-6)
Supplement: Supplementary file 1 — Supplementary information [file 41370_2025_762_MOESM1_ESM.docx]

**Supplemental Information**

# Associations Between Residential Fossil Fuel Combustion and Indoor Concentrations of Nitrogen Dioxide, Carbon Monoxide, and Aldehydes in Canadian Homes

Liu Sun^1^, Marie-Ève Héroux^1^, Xiaohong Xu^2^, Amanda J. Wheeler^3,4^

^1^ Water and Air Quality Bureau, Health Canada, Ottawa, Ontario, Canada

^2^ Department of Civil and Environmental Engineering, University of Windsor, Windsor, Ontario, Canada

^3^ Menzies Institute for Medical Research, University of Tasmania, Hobart, Tasmania, Australia

^4^ Commonwealth Scientific and Industrial Research Organization, Aspendale, Victoria, Australia

Table S1. Associations between indoor air pollutant concentrations and influencing factors. The table summarizes the geometric means and 95% confidence intervals for air pollutant concentrations, associated with per-unit changes in continuous variables or across categories of categorical variables.

| **Pollutant** | **Season** | **Factor** | **Category/Unit** | | **Geometric Mean Concentration of Pollutants and 95% Confidence Intervals** | | | | | | | | | | |
| --- | --- | --- | --- | --- | --- | --- | --- | --- | --- | --- | --- | --- | --- | --- | --- |
|  |  |  |  |  | **Edmonton** | | **Halifax** | | **Regina** | | **Windsor** | | **Pooled** | | |
| NO_2_ (µg/m^3^) | Summer | Presence of gas stoves | | Yes | | 12.64 (9.74-16.41) | | 10.92 (7.61-15.67) | | 13.16 (10.71-16.16) | | 20.66 (16.52-25.83) | | 17.16 (13.24-22.25) |  |
|  |  |  |  | No | | 9.64 (8.32-11.17) | | 3.88 (3.32-4.55) | | 5.84 (5.02-6.79) | | 8.67 (7.57-9.92) | | 8.03 (6.29-10.25) |  |
|  |  | Presence of gas clothes dryers | | Yes | |  | |  | |  | |  | | 14.23 (10.38-19.51) |  |
|  |  |  |  | No | |  | |  | |  | |  | | 9.68 (7.07-13.26) |  |
|  |  | Outdoor NO_2_ concentrations | | 10 % | | 1.52 (1.40-1.66) | | 1.77 (1.58-1.97) | | 1.26 (1.07-1.48) | | 2.42 (2.29-2.54) | | 2.23 (1.97-2.53) |  |
|  |  | Home construction year | | Before 1949 | | 14.71 (11.23-19.26) | | 8.86 (6.51-12.06) | |  | | 14.49 (11.21-18.73) | | 13.54 (10.30-17.79) |  |
|  |  |  |  | 1949 to 1990 | | 9.84 (8.01-12.09) | | 5.05 (3.85-6.62) | |  | | 15.08 (12.78-17.80) | | 11.60 (9.07-14.82) |  |
|  |  |  |  | After 1990 | | 9.30 (7.58-11.41) | | 6.17 (4.70-8.12) | |  | | 10.96 (8.84-13.59) | | 10.30 (8.02-13.23) |  |
|  |  | Windows opened | | Yes | | 12.64 (10.95-14.58) | |  | | 10.01 (8.66-11.57) | | 16.56 (13.75-19.96) | | 13.67 (10.66-17.53) |  |
|  |  |  |  | No | | 9.64 (7.85-11.84) | |  | | 7.68 (6.21-9.48) | | 10.81 (9.43-12.39) | | 10.08 (7.34-13.84) |  |
|  |  | Indoor relative humidity | | 1 % | |  | |  | | 1.02 (1.00-1.04) | |  | |  |  |
|  |  | Indoor temperature | | 1 ^°^C | |  | | 1.07 (1.02-1.12) | | 1.08 (1.02-1.15) | |  | |  |  |
|  | Winter | Presence of gas stoves | | Yes | | 26.93 (21.63-33.52) | | 22.61 (14.37-35.57) | |  | | 26.56 (15.97-44.18) | | 29.83 (16.01-55.55) |  |
|  |  |  |  | No | | 13.29 (11.81-14.96) | | 4.98 (3.36-7.38) | |  | | 8.79 (5.87-13.14) | | 10.27 (4.72-22.34) |  |
|  |  | Heating system fuel type | | Gas, homes built before 1949 | | 25.36 (20.61-31.21) | | 10.77 (4.47-25.99) | |  | | 23.19 (10.99-48.94) | | 21.86 (12.65-37.77) |  |
|  |  |  |  | Gas, homes built 1949 to 1990 | | 17.41 (14.42-21.03) | |  | |  | | 16.08 (10.80-23.92) | | 17.47 (9.82-31.09) |  |
|  |  |  |  | Gas, homes built after 1990 | | 15.33 (12.93-18.16) | | 10.47 (4.15-26.44) | |  | | 10.45 (4.40-24.81) | | 14.98 (8.04-27.89) |  |
|  |  |  |  | Oil | |  | | 14.41 (10.79-19.26) | |  | |  | | 21.31 (9.83-46.23) |  |
|  |  |  |  | Electricity | |  | | 7.80 (4.75-12.80) | |  | | 13.98 (7.59-25.75) | | 13.46 (7.42-24.42) |  |
|  |  | Presence of gas clothes dryers | | Yes | |  | |  | |  | |  | | 21.70 (12.47-37.75) |  |
|  |  |  |  | No | |  | |  | |  | |  | | 14.11 (3.79-52.54) |  |
|  |  | Candles used | | Yes | | 20.85 (17.73-24.52) | | 12.00 (8.39-17.17) | |  | |  | | 21.67 (11.33-41.46) |  |
|  |  |  |  | No | | 17.16 (15.23-19.33) | | 9.38 (6.76-13.02) | |  | |  | | 14.13 (6.35-31.44) |  |
|  |  | Outdoor NO_2_ concentrations | | 10 % | | 1.35 (1.28-1.42) | | 1.24 (1.13-1.35) | |  | | 1.41 (1.08-1.84) | | 1.52 (1.18-1.96) |  |
|  |  | Close to construction sources | | Yes | |  | | 14.03 (9.21-21.37) | |  | |  | | 20.68 (10.68-40.04) |  |
|  |  |  |  | No | |  | | 8.03 (5.57-11.57) | |  | |  | | 14.81 (6.77-32.37) |  |
|  |  | Indoor temperature | | 1 ^°^C | | 1.04 (1-1.09) | | 1.09 (1.01-1.17) | |  | |  | |  |  |
|  |  | Indoor relative humidity | | 1 % | |  | | 1.04 (1.01-1.07) | |  | |  | |  |  |
| Peak hourly CO (ppm) | Summer | Presence of gas stoves | | Yes | |  | |  | | 2.14 (1.88-2.44) | |  | |  |  |
|  |  |  |  | No | |  | |  | | 1.70 (1.54-1.87) | |  | |  |  |
|  |  | Candles used | | Yes | |  | | 1.42 (1.11-1.82) | |  | |  | |  |  |
|  |  |  |  | No | |  | | 1.26 (1.01-1.59) | |  | |  | |  |  |
|  |  | Windows opened | | Yes | | 3.51 (3.28-3.77) | |  | |  | |  | |  |  |
|  |  |  |  | No | | 3.74 (3.43-4.08) | |  | |  | |  | |  |  |
|  |  | Indoor relative humidity | | 1 % | | 1.02 (1.01-1.03) | | 0.99 (0.98-1.00) | |  | |  | |  |  |
|  |  | Indoor temperature | | 1 ^°^C | | 1.08 (1.07-1.10) | | 1.03 (1.00-1.05) | | 1.03 (1.01-1.05) | |  | | 1.05 (1.04-1.07) |  |
|  |  | Air exchange rate | | 1 h^-1^ | |  | | 0.83 (0.76-0.91) | |  | |  | | 0.95 (0.90-1.00) |  |
|  | Winter | Presence of gas stoves | | Yes | | 3.42 (2.56-4.56) | |  | |  | |  | | 2.92 (2.13-4.01) |  |
|  |  |  |  | No | | 2.21 (1.91-2.55) | |  | |  | |  | | 2.04 (1.79-2.32) |  |
|  |  | Indoor temperature | | 1 ^°^C | | 1.11 (1.06-1.16) | | 1.06 (0.99-1.13) | |  | |  | | 1.04 (1.01-1.07) |  |
| Daily average CO (ppm) | Summer | Presence of attached garages | | Yes | |  | |  | | 1.62 (1.45-1.81) | |  | |  |  |
|  |  |  |  | No | |  | |  | | 1.36 (1.21-1.52) | |  | |  |  |
|  |  | Indoor relative humidity | | 1 % | | 1.01 (1.00-1.02) | | 0.99 (0.99-1.00) | |  | |  | |  |  |
|  |  | Indoor temperature | | 1 ^°^C | | 1.07 (1.06-1.08) | | 1.02 (1.00-1.04) | | 1.03 (1.02-1.05) | |  | | 1.05 (1.03-1.06) |  |
|  |  | Air exchange rate | | 1 h^-1^ | | 0.91 (0.86-0.95) | |  | |  | |  | | 0.95 (0.92-0.98) |  |
|  | Winter | Presence of attached garages | | Yes | | 2.02 (1.75-2.33) | |  | |  | |  | |  |  |
|  |  |  |  | No | | 1.70 (1.51-1.92) | |  | |  | |  | |  |  |
|  |  | Indoor temperature | | 1 ^°^C | | 1.06 (1.03-1.10) | |  | |  | |  | | 1.03 (1.01-1.05) |  |
|  |  | Indoor relative humidity | | 1 % | |  | |  | |  | |  | | 1.01 (1.00-1.02) |  |
| Formaldehyde (µg/m^3^) | Summer | Home construction year | | Before 1949 | | 15.51 (11.56-20.81) | | 26.38 (19.03-36.58) | | 33.02 (21.03-51.85) | |  | | 25.32 (10.37-61.86) |  |
|  |  |  |  | 1949 to 1990 | | 25.54 (21.32-30.60) | | 29.35 (22.70-37.95) | | 30.48 (22.52-41.26) | |  | | 32.43 (10.94-96.12) |  |
|  |  |  |  | After 1990 | | 31.88 (26.28-38.68) | | 49.81 (37.24-66.64) | | 40.11 (29.85-53.90) | |  | | 48.39 (18.64-125.65) |  |
|  |  | Major renovations within the last month | | Yes | |  | | 42.10 (28.47-62.25) | | 44.03 (26.53-73.06) | |  | | 46.21 (21.78-98.01) |  |
|  |  |  |  | No | |  | | 27.12 (23.20-31.70) | | 26.73 (21.46-33.30) | |  | | 25.20 (4.92-129.07) |  |
|  |  | Hair spray used | | Yes | |  | | 35.57 (28.34-44.65) | |  | |  | |  |  |
|  |  |  |  | No | |  | | 32.09 (25.94-39.70) | |  | |  | |  |  |
|  |  | Windows opened | | Yes | | 22.28 (19.42-25.57) | | 31.20 (25.26-38.52) | | 29.41 (21.38-40.47) | |  | | 29.00 (7.39-113.74) |  |
|  |  |  |  | No | | 24.34 (21.01-28.20) | | 36.60 (29.05-46.10) | | 40.02 (29.13-54.97) | |  | | 40.16 (18.24-88.39) |  |
|  |  | Air cleaner used | | Yes | |  | |  | | 25.04 (16.46-38.1) | |  | |  |  |
|  |  |  |  | No | |  | |  | | 46.99 (36.00-61.35) | |  | |  |  |
|  |  | Presence of dehumidifiers | | Yes | |  | | 28.30 (22.07-36.28) | |  | |  | | 29.02 (11.30-74.53) |  |
|  |  |  |  | No | |  | | 40.34 (30.91-52.65) | |  | |  | | 40.13 (14.02-114.89) |  |
|  |  | Air conditioning used | | Yes | | 22.07 (18.61-26.16) | |  | | 38.94 (28.15-53.87) | |  | |  |  |
|  |  |  |  | No | | 24.58 (21.58-28.00) | |  | | 30.22 (22.25-41.05) | |  | |  |  |
|  |  | Air exchange rate | | 1 h^-1^ | | 0.56 (0.52-0.60) | | 0.59 (0.56-0.63) | |  | |  | | 0.49 (0.41-0.58) |  |
|  |  | Indoor relative humidity | | 1 % | | 1.02 (1.01-1.03) | | 1.01 (1.00-1.02) | |  | |  | |  |  |
|  |  | Indoor temperature | | 1 ^°^C | | 1.08 (1.06-1.10) | | 1.12 (1.09-1.14) | |  | |  | | 1.06 (1.01-1.10) |  |
|  | Winter | Home construction year | | Before 1949 | | 15.66 (12.82-19.14) | | 15.85 (12.07-20.82) | | 14.33 (11.45-17.95) | |  | | 15.83 (10.19-24.58) |  |
|  |  |  |  | 1949 to 1990 | | 22.10 (18.87-25.87) | | 18.77 (14.47-24.34) | | 21.91 (20.15-23.82) | |  | | 21.23 (12.65-35.62) |  |
|  |  |  |  | After 1990 | | 25.41 (21.71-29.74) | | 35.26 (27.48-45.24) | | 31.25 (26.45-36.91) | |  | | 29.20 (18.13-47.03) |  |
|  |  | Indoor relative humidity | | 1 % | | 1.03 (1.02-1.04) | | 1.05 (1.04-1.06) | | 1.04 (1.03-1.05) | |  | | 1.04 (1.02-1.05) |  |
|  |  | Indoor temperature | | 1 ^°^C | | 1.09 (1.07-1.12) | | 1.10 (1.08-1.13) | | 1.10 (1.06-1.14) | |  | | 1.08 (1.04-1.11) |  |
|  |  | Air exchange rate | | 1 h^-1^ | | 0.77 (0.63-0.93) | | 0.65 (0.47-0.90) | |  | |  | | 0.71 (0.50-1.02) |  |
| Acetaldehyde (µg/m^3^) | Summer | Home construction year | | Before 1949 | | 5.31 (3.34-8.46) | | 4.43 (3.04-6.46) | |  | |  | | 4.95 (3.72-6.59) |  |
|  |  |  |  | 1949 to 1990 | | 8.84 (6.56-11.91) | | 5.92 (4.40-7.97) | |  | |  | | 7.26 (5.92-8.92) |  |
|  |  |  |  | After 1990 | | 9.03 (6.55-12.45) | | 7.07 (4.98-10.03) | |  | |  | | 8.09 (6.42-10.18) |  |
|  |  | Perfume used | | Yes | |  | |  | | 10.00 (7.58-13.20) | |  | |  |  |
|  |  |  |  | No | |  | |  | | 8.14 (6.40-10.35) | |  | |  |  |
|  |  | Air conditioning used | | Yes | |  | |  | | 11.86 (9.20-15.30) | |  | |  |  |
|  |  |  |  | No | |  | |  | | 6.86 (5.28-8.92) | |  | |  |  |
|  |  | Windows opened | | Yes | |  | |  | | 7.23 (5.61-9.31) | |  | |  |  |
|  |  |  |  | No | |  | |  | | 11.26 (8.58-14.77) | |  | |  |  |
|  |  | Air cleaner used | | Yes | |  | |  | | 6.99 (4.52-10.82) | |  | |  |  |
|  |  |  |  | No | |  | |  | | 11.64 (10.22-13.27) | |  | |  |  |
|  |  | Air exchange rate | | 1 h^-1^ | | 0.88 (0.78-1) | | 0.89 (0.82-0.96) | |  | |  | | 0.87 (0.81-0.94) |  |
|  | Winter | Home construction year | | Before 1949 | | 6.89 (5.15-9.21) | | 4.15 (2.59-6.65) | | 6.98 (4.63-10.52) | |  | | 6.25 (2.52-15.46) |  |
|  |  |  |  | 1949 to 1990 | | 10.70 (8.52-13.44) | | 4.42 (2.82-6.91) | | 8.59 (7.36-10.04) | |  | | 7.31 (2.65-20.14) |  |
|  |  |  |  | After 1990 | | 9.41 (7.49-11.82) | | 10.65 (6.93-16.37) | | 13.28 (9.85-17.9) | |  | | 9.47 (3.60-24.92) |  |
|  |  | Cooked with oil | | Yes | |  | |  | | 10.60 (8.37-13.41) | |  | |  |  |
|  |  |  |  | No | |  | |  | | 8.11 (6.60-9.95) | |  | |  |  |
|  |  | Indoor relative humidity | | 1 % | |  | | 1.03 (1.01-1.05) | | 1.05 (1.03-1.07) | |  | | 1.04 (1.02-1.06) |  |
|  |  | Indoor temperature | | 1 ^°^C | |  | |  | | 1.10 (1.03-1.17) | |  | | 1.06 (1.01-1.10) |  |
|  |  | Air exchange rate | | 1 h^-1^ | | 0.78 (0.59-1.03) | |  | |  | |  | | 0.42 (0.26-0.69) |  |


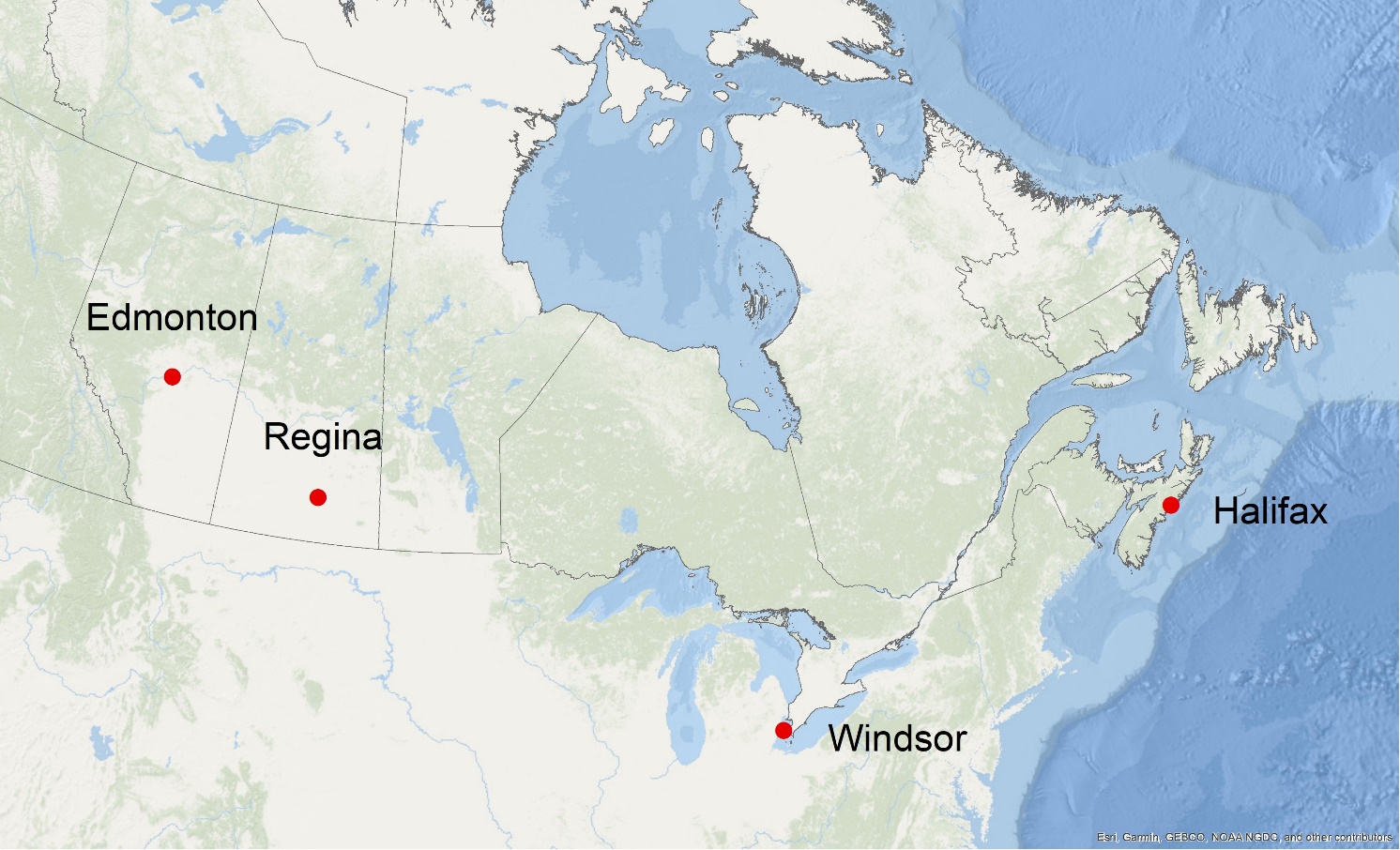


**Figure S1. Locations of the four study cities across Canada.**
